# Examining characteristics of recorded and unrecorded alcohol consumers in Kenya

**DOI:** 10.1186/s12889-018-5960-1

**Published:** 2018-08-23

**Authors:** Rahma S. Mkuu, Adam E. Barry, Francisco A. Montiel Ishino, Ann O. Amuta

**Affiliations:** 1 Division of Health Education, Department of Health & Kinesiology, Texas A&M University, 4243 TAMU, Blocker Building, Office 311G, College Station, TX 77843-4243 USA; 2 Department of Health Studies, Texas Woman’s University, 304 Administration Dr. CFO 1011, Denton, TX 75204 USA

**Keywords:** Alcohol, Unregistered alcohol, Unrecorded alcohol, Homebrew, Kenya, Alcohol policy

## Abstract

**Background:**

Due to media reports of several deaths, consumption of unrecorded alcohol (i.e., alcohol brewed at home) has emerged as a public health threat in developing countries like Kenya. Empirical data on this issue, however, is scarce. This investigation compared demographic characteristics of Kenyans who drank recorded (regulated) and unrecorded alcohol.

**Methods:**

We examined all respondents who consumed alcohol in the past month (*N* = 718) on the 2015 nationally representative Kenya STEPwise survey. Descriptive statistics and bivariate logistic regression examined proportion of respondents consuming unrecorded alcohol, and social demographic factors associated with unrecorded alcohol consumption, respectively.

**Results:**

The sample was primarily male (86%), married (64%), middle class or higher (64%), with an average age of 37 years. Participants reported an average of 2.5 drinking events and 4.3 binge-drinking occasions per month. Overall, 37% of our sample consumed unrecorded alcohol. Compared to those with incomplete primary education or lower, individuals who completed primary education or above were less likely to report consuming unrecorded alcohol (OR = 0.22, 95% CI: 0.12–0.43). Compared to poorest and poor respondents, those identifying as middle class or above were less likely to consume unrecorded alcohol (OR = 0.47, 95% CI: 0.29–.78). Current smokers (OR = 2.19, 95% CI: 1.34–3.60) and those with higher binge drinking occasions in the past month (OR = 1.03, 95% CI: 1.004–1.07) were significantly more likely to consume unrecorded alcohol.

**Conclusion:**

Kenyan adults who consume unrecorded alcohol engage in more binge drinking occasions, smoke, and have lower levels of education and socioeconomic status. It is vital that health promotion interventions aimed at reducing unrecorded alcohol consumption be tailored and targeted to individuals with low socio-economic status in Kenya.

## Background

Alcohol consumption contributes to over 5% of the global burden of disease and is ranked among the leading risk factors for morbidity and mortality worldwide [1]. Consumption is associated with cardiovascular disease and increased rates of exposure to HIV infection, tuberculosis, and pneumonia, which are among the global leading causes of death and disability [2–5]. Intentional and unintentional injuries are also associated with alcohol consumption [6–8].

Across the globe, there are two primary types of alcohol consumed, recorded and unrecorded. Recorded alcohol refers to alcohol that is regulated, controlled, tracked, and legally purchased [9]. On the other hand, unrecorded alcohol is alcohol that is not regulated and is: (1) illegally produced; (2) illegally imported through cross-border smuggling; (3) homebrew/homemade; (4) not consumed where it is registered, e.g., duty free shops; or (5) not intended for consumption, i.e., surrogate alcohol such as aftershave [10–12]. It is estimated that 25% of alcohol consumed globally is unrecorded [13] and consumption is associated with many unique risks. Unrecorded alcohol consumption represents a unique public health threat worthy of further investigation.

Compared to recorded alcohol, unrecorded alcohol in sub-Saharan Africa is consumed at more frequency and in greater quantities [14]. For instance, in Kenya unrecorded alcohol is consumed more frequently than recorded alcohol [15–17]. In Kenya, several public health concerns are associated with unrecorded alcohol consumption, such as fatalities and hospitalizations [18–20]. On May 7th 2014, it was reported that over 75 people in Kenya died and 181 were hospitalized as a result of consuming unrecorded alcohol [21].

Many of the deleterious consequences associated with unrecorded alcohol consumption in Kenya are attributed to the practice of adulterating the alcohol with harmful substances such as methanol, car battery acid, and other harmful substances believed to increase potency [16] . For example, *chang’aa* a distilled homebrew unrecorded alcohol in form of a spirit (unsweetened distilled alcohol with higher alcohol content) in Kenya is nicknamed “kill me quick” for its high perceived and actual measured potency [16, 22]. A unique characteristic of unrecorded alcohol in Kenya is those who brew also commercialize it. The unrecorded alcohols such as homebrew which are typically sold from homes are also generally cheap and affordable [15, 23]. A glass of *chang’aa* is 50 Kenyan shillings ($0.50 United States (U.S.)) compared to a bottle of recorded beer that costs twice as much [24]. Additionally, unrecordedalcohol in Kenya is closely tied to traditional and cultural practices [15]. Due to this constellation of factors - high potency, low cost, and cultural importance – consumption of unrecorded alcohol in Kenya continues to be high and significantly contributes to the burden of morbidity and mortality in the country.

The health and social challenges of recorded alcohol consumption are relatively well established; however, there is a significant gap in the literature examining unrecorded alcohol consumption, particularly in Africa. To the best of our knowledge, there is a scarcity of studies specifically examining consumption patterns and demographic characteristics of recorded and unrecorded alcohol consumption in Kenya [25, 26]. Consequently, herein we examined: (1) differences in number of drinking events in the last month and binge drinking defined as having 6 standard drinks during at least one occasion in the last 30 days [13] between individuals who exclusively consumed recorded alcohol and those who reported consuming unrecorded alcohol; and (2) demographic differences between individuals who exclusively consumed recorded alcohol and those who consumed unrecorded alcohol.

## Methods

### Sample

We examined secondary cross-sectional data from the nationally representative 2015 Kenya World Health Organization (WHO) STEPwise Survey [27]. The nationally representative survey employed a three-stage cluster sampling design whereby clusters were selected to evenly represent rural and urban areas [27]. The clusters were selected by region (rural vs. urban), then by households and finally by individuals. A total of 4500 eligible adults, ages 18–64, participated in the survey with a response rate of 95% [27]. More details on sampling and methods are provided in the 2015 Kenya WHO STEPwise report [27]. For the purposes of this research, only participants who consumed alcohol within the last 30 days (*N* = 718) were included.

#### Dependent variables

Type of alcohol consumed (recorded vs. unrecorded) was assessed using the following question: “During the past 7 days, did you consume any homebrewed alcohol (including *chang’aa*, *busaa,* or *muratina*) or any alcohol not intended for drinking?” Participants who responded “no” were identified as recorded alcohol consumers. Participants who responded “yes” were identified as recent homebrew/unrecorded alcohol consumers.

#### Covariates

*Binge drinking* was defined as consuming more than 6 standard drinks per drinking occasion [13]. Participants were asked to recall the number of drinking occasions that they consumed 6 or more standard drinks in the last 30 days. A standardized drink was defined as the amount of alcohol in a small beer, one glass of wine, or one tot of spirits [27].

Participant demographic variables included gender (i.e., male, female), age (ranging from 18 to 64 years), marital status (i.e., cohabitating, currently married, divorced, never married, separated, and widowed), residence (i.e., urban, rural), region (i.e., Rift Valley, Western, Nyanza, Central, Coast, Eastern, North Eastern, and Nairobi), and wealth index (i.e., poorer, poor, middle, rich and richest). Current smoking status (i.e., yes, no) was included to explore association with risk behaviors. Number of drinking occasions was defined as the number of occasions participants consumed at least one standard alcoholic drink within the last 30 days*.*

#### Statistical analysis

StataCorp Stata Statistical Software for Windows (Version 14.2, College Station, Texas, U.S) was used to analyze the data. The Stata survey procedures were applied to analyze the data after considering the survey stratification and clustering design variables and sample weights to ensure the correct estimation of sampling error.

Frequencies for categorical and binary variables and means and standard deviations for continuous variables were calculated to provide descriptive statistics of variables. Binary logistic regression was conducted to predict the relationship between alcohol consumption type (recorded versus unrecorded) and number of drinking occasions/events in the past month and binge drinking occasions in the past month, controlling for demographic characteristics**.**

## Results

### Demographic characteristics

The mean age of participants was 36.6 years. A majority of the sample were male 86%, married (64%) and middle class or higher (64%). Overall, 29% of respondents were current smokers and among the alcohol consumers, 37% consumed unrecorded alcohol. The mean number of drinking events in the past month was 2.6 occasions and the mean number of binge drinking events in the past month was 4.3. Table 1 provides descriptive statistics for the sample.

**Table 1:** Demographic characteristics of the sample (*n* = 718) (to be presented in page 6)

	Proportion	SE	95% CI	
Sex				
Male	85.71%	0.02	0.81	0.90
Female	14.29%	0.02	0.10	0.19
Age in years				
18–29	35.45%	0.03	0.29	0.42
30–44	41.22%	0.03	0.36	0.47
45–59	16.61%	0.02	0.13	0.21
60–69	6.72%	0.01	0.05	0.10
Marital Status				
Cohabitating	1.00%	> 0.003	> 0.003	0.03
Married	64.42%	0.03	0.59	0.69
Divorced	0.08%	> 0.003	>.003	0.02
Never Married	20.46%	0.03	0.16	0.26
Separated	8.68%	0.02	0.06	0.13
Widowed	4.75%	0.02	0.02	0.09
Education				
No formal Schooling	7.10%	0.02	0.04	0.12
Primary Incomplete	29.59%	0.04	0.22	0.38
Primary Complete	24.86%	0.03	0.19	0.31
Secondary and above	38.45%	0.06	0.28	0.50
Wealth Index				
Poorest	17.97%	0.04	0.12	0.26
Poor	18.06%	0.03	0.14	0.24
Middle	16.65%	0.02	0.12	0.22
Richer	18.42%	0.04	0.11	0.28
Richest	28.90%	0.06	0.19	0.42
Residence				
Rural	54.48%	0.05	0.44	0.65
Urban	45.52%	0.05	0.35	0.56
Region				
Nairobi	12.81%	0.06	0.04	0.32
Rift Valley	26.36%	0.05	0.18	0.37
Western	13.10%	0.04	0.07	0.22
Eastern	13.40%	0.04	0.08	0.22
Central	14.22%	0.05	0.07	0.27
Nyanza	9.96%	0.03	0.06	0.17
Coast	10.14%	0.03	0.05	0.19
Homebrew Consumers	37.37%	0.05	0.28	0.47
Current Smoker	29.35%	0.03	0.24	0.35
Binge Drinkers	70.22%	0.03	0.64	0.76
	Mean	SE	95% CI	
Age	36.64	0.74	35.18	38.11
# Drinking Occasions past month	2.56	0.11	2.33	2.78
# Binge Drinking Occasions past month	4.27	0.48	3.32	5.22

Among participants who reported consuming unrecorded alcohol, the mean age was 38.5 years compared to 35.7 years among non-consumers (individuals who did not consume unrecorded alcohol). The sex make up for both unrecorded consumers and non-consumers was comparable (86% compared to 85%). A slightly larger proportion of unrecorded consumers were married (67%), compared to con-consumers, (63%). Majority of unrecorded alcohol consumers did not complete primary education (61%), while (22%) of non-consumers did not complete primary education. A larger proportion of unrecorded consumers were of the lowest economic categories; poorest (27%) or poor (27%), compared to non-consumers (13% and 15% respectively). More than half of the unrecorded alcohol consumers resided in rural areas (67%) compared to (47%) of non-consumers residing in rural areas. Furthermore, (73%) of unrecorded alcohol consumers were identified as binge drinkers compared to (69%) of non-consumers. Homebrew beer or wine were the most consumed unrecorded alcohol type (mean = 6.3 drinks) followed by homebrew spirits (mean = 1.9 drinks) reported by unrecorded consumers. Table 2 compares descriptive statistics of unrecorded alcohol consumers and those who did not report consuming unrecorded alcohol (ie. Consumed only recorded alcohol).

**Table 2:** Sample descriptive statistics for unrecorded alcohol consumers compared to non-consumers (n = 718) (to be presented in page 7)

	Proportion Unrecorded			Proportion Recorded		
Sex						
Male	86.42%			85.29%		
Female	13.58%			14.71%		
Age in years						
18–29	27.58%			40.15%		
30–44	46.22%			38.24%		
45–59	17.16%			16.28%		
60–69	9.03%			5.34%		
Marital Status						
Cohabitating	1.29%			0.70%		
Married	67.47%			62.61%		
Divorced	0.37%			1.01%		
Never Married	14.19%			24.19%		
Separated	6.24%			10.13%		
Widowed	10.44%			1.35%		
Education						
No Formal Schooling	10.98%			4.80%		
Primary Incomplete	50.25%			17.25%		
Primary Complete	25.96%			24.20%		
Secondary and above	12.80%			53.76%		
Wealth Index						
Poorest	27.01%			12.58%		
Poor	27.18%			12.62%		
Middle	19.62%			14.88%		
Rich	20.82%			16.99%		
Richest	5.37%			42.93%		
Residence						
Rural	67.10%			46.95%		
Urban	32.90%			53.05%		
Region						
Nairobi	10.97%			13.90%		
Rift Valley	30.75%			23.74%		
Western	20.24%			8.86%		
Eastern	14.21%			12.92%		
Central	1.00%			22.11%		
Nyanza	13.02%			8.13%		
Coast	9.81%			10.33%		
Smoker						
No	58.87%			77.68%		
Yes	41.13%			22.32%		
Binge Drinking (w/in last month)						
No	27.29%			31.27%		
Yes	72.71%			68.73%		
	Mean	95% CI		Mean	95% CI	
Age	38.45	36.57	40.33	35.66	33.80	37.52
# Drinking Occasions (past month)	2.61	2.30	2.92	2.53	2.24	2.81
# Binge Drinking (occasions past month)	5.66	4.12	7.21	3.46	2.32	4.61

The logistic regression model demonstrated that individuals who consumed unrecorded alcohol were significantly more likely to binge drink, have less than primary education, be in the poorest or poor wealth category, and be current smokers. Results demonstrated that unrecorded alcohol consumption was significantly associated with having higher binge drinking occasions compared to recorded alcohol consumption, after controlling for demographic characteristics and number of drinking occasions in the past month (OR: 1.03, 95% CI: 1.004–1.07). Participants who completed primary education or above compared to individuals who did not complete primary education had lower odds of consuming unrecorded alcohol (OR = 0.22, 95% CI: 0.12–0.43). Compared to poor respondents, participants identifying as middle class or above were less likely to consume unrecorded l alcohol (OR = 0.47, 95% CI: 0.29–0.78). Current smokers (OR: 2.19, 95% CI: 1.34–3.60) were significantly more likely to consume unrecorded alcohol. Results from the logistic regression are outlined in Table 3.

**Table 3:** Estimates of odds ratios and confidence intervals of correlates of unrecorded alcohol consumption among individuals who consumed alcohol within the last month

Variables	OR	95% Conf. Interval	
		Lower	Upper
Age	1.01	0.99	1.03
Sex	0.83	0.48	1.45
Marital Status	1.07	0.90	1.26
Education (Primary and Above)	0.22	0.12	0.43
Economic Status (Middle Class and Above)	0.47	0.29	0.78
Rural	1.33	0.63	2.83
Region	1.16	0.97	1.38
Current Smoker	2.19	1.34	3.60
Number occasions consumed alcohol last month	1.01	0.89	1.14
Binge drinking events last month	1.03	> 1.00	1.07
Constant	0.54	0.12	2.36

An interaction model between level of education (completed primary education or above and less than primary education) and wealth index (poor and poorer vs. middle to richest) demonstrated that participants with completed primary education, and were middle class or above, were significantly less likely to be unrecorded alcohol consumers (OR = 0.18, 95% CI: 0.06–0.51). Results of the interaction model are outlined in Table 4.

**Table 4:** Interaction model results between education and economic status

Variables	OR	95% Conf. Interval	
		Lower	Upper
Education (Primary and Above)	0.59	0.33	1.08
Economic Status (Middle Class and Above)	1.17	0.46	2.96
Education*Economic Status	0.18	0.06	0.51
Constant	1.56	1.00	2.44

*means an interaction effect between education and economic status and how the two influence unrecorded alcohol consumption

## Discussion

The aim of this paper was to examine demographic differences between individuals who report consuming recorded alcohol and those who report recent unrecorded alcohol consumption in a nationally representative sample of Kenyan adults. Among individuals who reported consuming alcohol within the last month, those who reported recent consumption of unrecorded alcohol, were significantly more likely to engage in higher binge drinking occasions and more likely to be current smokers. We also found that individuals who reported unrecorded alcoholconsumption were more likely to have lower than a primary education, and were in the poor or poorest wealth categories thus of lower socio-economic status (SES) (i.e., poor and poorest wealth category).

Alcohol consumption, both recorded and unrecorded were higher among men. These findings are similar to prior research findings that report higher alcohol consumption prevalence among Kenyan men [28–31]. Olack and colleagues assert men are four times more likely to report consuming alcohol in the past thirty days compared to women in Nairobi, Kenya [28]. Excessive drinking in men poses a great public health concern as men are more likely than women to take behavioral risks, such as driving over the speed limit, not wearing a safety belt while operating a motor vehicle, and engage in physical altercations than women. Drinking excessively further increases the likelihood of injury, hospitalization, or death among men [32]. Collectively, these findings highlight the need for development and evaluation of gender-based public health initiatives to minimize the risks associated with alcohol consumption in Kenya.

Seventy seven percent of those reporting recent alcohol consumption were mostly young adults between 18 and 44 years. Consistent with our findings of high alcohol consumption among this age range, Jenkins and colleagues [30] and The National Campaign Against Drug Abuse Authority [20] found that young adults make up the largest proportion of individuals who consume alcohol. Overall, participants in our investigation study were evenly distributed by social class (i.e., poorest, poor, middle, richer, and richest), level of education (i.e., primary incomplete, primary complete, secondary or more), residence (i.e., rural versus urban), which may indicate how entrenched alcohol consumption is culturally in Kenya, given it was common across social and economic lines and geographic locations [30].

Our findings, when controlling for demographic characteristics and number of drinking occasions, revealed that unrecorded alcohol consumers were significantly more likely to report binge drinking occasions. Given the characteristic of unrecorded alcohol (i.e., low cost and high potency), our results lend credence to the notion that unrecorded alcohol consumption may lead to higher levels of alcohol consumed and result in greater alcohol-related consequences [13, 15]. While our investigation cannot tease out temporal associations, previous research has asserted individuals who consumed unrecorded alcohol are high-volume alcohol consumers [11], and consumption of unrecorded alcohol may contribute to heavy/high volume drinking [33].

Several demographic characteristics were also related to consumption of unrecorded alcohol. Education was found to be a protective factor for unrecorded alcohol consumption. Individuals who had not completed primary education had higher odds of consuming unrecorded alcohol. Our findings align with a previous study from Kinoti, Jason, and Harper [31] who contend individuals with lower education levels (52.5%) were significantly more likely to consume unrecorded alcohol compared to those with high education (37.8%). Similarly, individuals living in poverty, those in the poor or poorest wealth categories, were also more likely to report recent unrecorded alcohol consumption. Our findings reveal that social determinants leading to disparate health outcomes are present and persistent in the high prevalence of unrecorded alcohol consumption [28, 34].

Deaths from unrecorded alcohol consumption have been reported in low-income neighborhoods such as slums and rural areas in Kenya [21, 24, 35]. For example, 70 deaths from one tainted batch of unrecorded alcohol in a Slum in Nairobi Kenya has been reported [36]. An interaction effect between education and wealth was significant in our study, meaning individuals who had low education status were also poor and both groups significantly consumed more unrecorded alcohol. Those with low education may be more likely to have limited knowledge on the effects and dangers of drinking unrecorded alcohol [37]. In addition, due to the low cost of unrecorded alcohol, it is more likely to be consumed by individuals of low SES [23, 38, 39]. A study conducted among slum dwellers in Nairobi found that 50.3% of alcohol reported to be consumed was unrecorded homebrew [28]. Our results align with evidence of higher consumption of unrecorded alcohol such as homebrew in low and middle-income settings [13, 40].

Public health interventions aimed at monitoring and evaluating the effects and consumption of unrecorded alcohol should target low SES individuals and those with lower levels of educational attainment. Health promotion programs should also work to educate individuals on the negative effects of unrecorded alcohol [37]. In Kenya, homebrew was legalized in 2013–2014 under the condition that brewers register and follow guidelines to ensure safe brew processes. However, after cases of methanol poisoning were reported from unregistered brewers in 2014, homebrew was outlawed again. There has yet to be evidence of whether the policy is improving and reducing negative effects such as mortality from homebrew.

While results from this study provides provide additional insights into the context of unrecorded alcohol consumption in Kenya, there are several limitations that should be considered. First, the data was cross-sectional, thus limiting the ability to analyze trends over time. Second, because the data were of a secondary nature, the variables available were limited. For example, unrecorded alcohol consumption was measured using a question asking about whether participants consumed homebrew within the last week. It is recommended that questions regarding alcohol consumption assess one’s pattern of drinking which can differentiate between irregular consumption of large quantities, and regular consumption of small quantities [41]. Thus, future investigations would benefit from more nuanced assessments of unrecorded alcohol consumption. Third, our study was disproportionally male; however, the overrepresentation of males is supported by evidence of higher prevalence of alcohol consumption among men in Kenya.

## Conclusions

Given this is one of the first, or few, nationally representative investigations examining differences between individuals who consume recorded alcohol and those who consume unrecorded alcohol in Kenya, our study adds to the scarce literature on unrecorded alcohol consumption behaviors and determinants in Kenya. Overall, our study found that individuals who consume unrecorded alcohol are more likely to binge drink and smoke. These individuals are from lower SES and are more likely to have low levels of education. These demographic factors provide an initial evidence base for the beginning formation of educational and social marketing efforts targeting unrecorded alcohol consumers. Our focus on unrecorded alcohol consumption should not be interpreted as indication that recorded alcohol is less harmful than unrecorded alcohols such as homebrew. We recognize alcohol consumption is one of the leading risk factors for morbidity and mortality worldwide and both unrecorded alcohol such as homebrew and recorded alcohols such as commercialized beer are both public health concerns. That said, unrecorded alcohol consumption is unique, and will consequently require novel investigations and interventions. Our study bridges an important literature gap; understanding unrecorded alcohol consumption behaviors and the socio-demographic factors that are unique to individuals who consume unrecorded alcohol. Targeted and tailored interventions to reduce unrecorded alcohol consumption should focus on individuals who are current smokers, poor, and those with low educational attainment.

## Abbreviations

CI: Confidence Intervals
HIV: Human Immunideficiency Virus
OR: Odds Ratio
SE: Standard Error
SES: Socio-economic status
U.S.: United States
